# Individual Differences in Plate Wasting Behavior: The Roles of Dispositional Greed, Impulsivity, Food Satisfaction, and Ecolabeling

**DOI:** 10.3390/bs13080626

**Published:** 2023-07-27

**Authors:** Engin Üngüren, Ömer Akgün Tekin, Hüseyin Avsallı, Yaşar Yiğit Kaçmaz

**Affiliations:** 1 Department of Business Management, Faculty of Economics, Administrative and Social Sciences, Alanya Alaaddin Keykubat University, Antalya 07450, Türkiye; engin.unguren@alanya.edu.tr; 2 Department of Gastronomy and Culinary Arts, Manavgat Faculty of Tourism, Akdeniz University, Antalya 07600, Türkiye; 3 Department of Tourism Management, Graduate School of Education, Alanya Alaaddin Keykubat University, Antalya 07450, Türkiye; yykacmaz@hotmail.com

**Keywords:** food waste, plate waste, dispositional greed, impulsivity, food satisfaction, ecolabels

## Abstract

This study examines the effects of dispositional greed, impulsivity, food satisfaction, and ecolabeling on consumers’ plate waste in all-inclusive hotels. Using a moderated mediation research model, a cross-sectional survey was conducted among 1253 tourists of different nationalities, all staying in five-star hotels in Alanya, Türkiye. The results show that both dispositional greed and impulsivity positively predict and significantly contribute to plate waste. Conversely, food satisfaction was found to be an influential variable that moderates the effects of greed and impulsivity on plate waste, highlighting its critical role in waste reduction strategies. Ecolabels, despite their intended purpose, were not found to have a significant impact on consumer attitudes toward plate waste. Future research is encouraged to explore strategies to counteract dispositional greed and impulsivity, given their significant impact on plate waste behavior. At the same time, refining methods to promote food satisfaction and the effective use of ecolabels may contribute significantly to reducing plate waste in all-inclusive resorts. This research contributes to our understanding of the psychological factors that influence consumer behavior in buffet settings and provides guidance to hospitality industry practitioners seeking to reduce waste.

## 1. Introduction

As vital factors for the global economy to survive, consumption and production can have destructive impacts on our planet [1]. From this point of view, United Nationals determined 17 Sustainable Development Goals in 2015, including “Goal 12: Ensure sustainable consumption and production patterns” to build a sustainable future [2]. Sustainable consumption and production (SCP) focus on doing more and better by using less, reducing poverty, and contributing to switching low-carbon and green economies [1]. In this context, “food” is one of the sub-headings of SCP. Large amounts of water, energy, and chemicals are used for food production [3]. Despite such exhaustion of resources, produced food is not always eaten, in addition to taking up too much space and producing CO_2_, all of which are harmful to the environment [4]. Wasting 1 kg of food, for example, produces 1.9 kg of CO_2_ [5]. Thus, even a 38% reduction in food waste is able to reduce its negative effects on climate by 40% and its negative effects on biodiversity by 30% [6]. On the other hand, one-third of all food that is produced, which is estimated to cost USD 1 trillion, becomes inedible due to several reasons [1]. Consequently, about 8–10% of global greenhouse gas emission is caused by food loss and food waste [7].

The UNEP Food Waste Index Report 2021 estimates that 931 million tons of food waste is generated globally each year. Of this, 61% comes from households, 26% from food service, and 13% from retail [8]. This amount of waste not only represents a significant economic loss but also contributes to environmental degradation [9]. This study focuses on the problem of plate waste, which is a critical type of food waste in the tourism and hospitality sector [10]. Nearly 30% of food waste in the tourism and hospitality sector is caused by plate waste [11]. Despite the growing interest in food waste in the literature, detailed studies on plate waste are lacking [12]. A significant part of the existing studies focuses on food waste in households [13,14,15], business practices [16], and wasted quantities [17]. Another topic that is just as important as the quantitative results of food waste is understanding consumers’ psychological reasons for food waste, a topic that represents a gap in the literature [18]. With this in mind, our study seeks to examine the impact of dispositional greed and impulsivity on plate waste while also assessing the moderating role of food satisfaction and ecolabeling. It seeks to contribute to the literature by examining the psychological factors that potentially drive plate waste behavior, thus filling a critical research gap.

The Turkish Ministry of Agriculture and Forestry initiated a campaign called “Save Your Food” in May 2020 in the hospitality and food service industry to raise awareness of food waste [19]. The main goal of this campaign was to raise awareness among both the hospitality industry and consumers about the severity of food waste. In a study conducted on only 4.5% of the five-star hotels in Türkiye, the amount of food waste was found to be around 180 tons in only a month [20]. Considering that there are 861 five-star hotels in Türkiye as of 2023 [21], the food waste amounts in the tourism and hospitality sector are reaching alarming levels. Therefore, this study was conducted in hotels offering a buffet service system, which became a fundamental component of the all-inclusive holiday concept in Türkiye [22]. The buffet service system is an important source of plate waste because selections are presented in impressive ways and without portion limits [4,23,24].

This research aims to make a substantial contribution to the field of consumer psychology and waste behavior, particularly in the tourism and hospitality sector. Exploring the psychological aspects of consumers’ plate waste behavior provides a new dimension to understanding the problem of excessive food waste. First, from a theoretical perspective, our study aims to contribute to the body of knowledge by revealing the underlying psychological factors, namely greed and impulsivity, that drive plate waste behavior in buffet services. While the existing literature extensively examines the physical amounts of waste and the financial implications of such waste, few studies have addressed the root causes of such wasteful behaviors from a psychological perspective [12,18]. By integrating theories from consumer behavior and psychology, this study provides an in-depth examination of these dispositional factors and thus offers a comprehensive understanding of plate waste phenomena in the context of all-inclusive hotel services.

In addition, the research has practical implications, particularly for the hospitality industry. Understanding how food satisfaction and ecolabeling act as moderators in the relationship between dispositional traits (greed and impulsivity) and plate waste behavior could inform the development of interventions and strategies aimed at reducing plate waste. By demonstrating whether such strategies can effectively influence customer behavior to reduce plate waste, the results of this research could help hoteliers and policymakers formulate strategic plans that take into account consumers’ psychological characteristics. Furthermore, by potentially contributing to the reduction of plate waste, the study could assist the hospitality industry in achieving sustainable practices. Finally, on a broader scale, this research could have implications for studies of consumer behavior and sustainability. It is hoped that this research could stimulate further academic investigation into the psychological underpinnings of wasteful behavior, paving the way for a more comprehensive understanding of sustainable consumer practices.

## 2. Literature Review and Hypothesis Development

### 2.1. Plate Waste in the Hospitality Industry

Plate waste plays an important role in the generation of food waste [25,26] and is the main cause of food waste, particularly in the hospitality sector [27]. Studies in different regions have found disturbing levels of plate waste. According to Dolnicar [12], consumers leave about 11–13% of the food served on their plates as plate waste. Tomaszewska et al. [28], in a study they conducted to investigate the extent of food waste in hotels, found that 72.55% of the food served to consumers was wasted, while the average plate waste per plate was 5.8%. Another study by Tomaszewska et al. [29] also concluded that one in five consumers engaged in plate waste. Data from the Cornell College Food and Brand Lab shows that, on average, 17% of meals are left unfinished by diners [30], while a separate study conducted in the United Kingdom reported that 27% of consumers engage in plate waste [31]. In addition, the UK Sustainable Restaurant Association [32] concluded that consumer plate-wasting behavior accounts for 30% of food wasted in UK restaurants. The evidence from these various studies underscores the significant contribution of plate waste to the broader issue of food waste, particularly in the hospitality sector.

### 2.2. Factors Influencing Plate Waste in the Hospitality Sector

Under this heading, the factors affecting plate waste in the hospitality sector, which is the focus of our study, are discussed. Factors that influence plate waste can generally be divided into two categories: (1) organizational factors and (2) consumer factors. This review first outlines these two categories and then delves into each group, summarizing key findings from the relevant literature.

#### 2.2.1. Organization-Based Factors Influencing Plate Waste

Several elements within an organization can contribute to the problem of plate waste. For example, physical design aspects such as plate size and shape have been found to influence plate waste [33,34]. In addition, food- and service-related aspects such as food variety, portion size, and menu assortment also play an important role [25]. Certain sales policies aimed at stimulating consumption to increase revenue may inadvertently lead to increased plate waste [35]. In terms of hotel services, issues such as the provision of services to external guests, reservation coordination problems, and inconsistencies in food offerings have been reported to contribute to the waste problem [4]. In addition, factors related to the dining environment, such as the distance between tables and buffets, food quality, restaurant size, and restaurant occupancy, have been found to have a significant impact on plate waste [23,24,36,37]. Finally, operation-related aspects such as the lack of takeaway services for unconsumed food and errors in product planning, purchasing, delivery, storage, preparation, and cooking processes can also contribute to the generation of plate waste [38,39]. Each of these organization-related factors presents opportunities for intervention and waste reduction in the hospitality sector.

#### 2.2.2. Consumer-Based Factors Influencing Plate Waste

There are several consumer-related factors that can significantly contribute to the problem of plate waste. Consumers’ individual physiological states and food preferences, such as appetite and taste preferences, have been shown to affect the amount of waste produced [40]. Demographic characteristics, such as gender, nationality, and number of children, also play a role in this issue [24]. Other factors, such as personality traits and the purpose of the meal, may further exacerbate the problem of plate waste [41]. Behavioral elements are also important; the number of plates per person, the use of tobacco or alcohol during the meal, and dietary restrictions can also increase plate waste [35]. In addition, external influences, such as messages and events designed to alert consumers to the importance of reducing food waste, can have a significant impact on consumer behavior and, therefore, the amount of waste they produce [42]. Cultural factors can also influence plate waste. For example, food consumption practices, whether influenced by tradition or emerging food trends, can contribute to the overall level of plate waste [4]. This includes phenomena such as food neophilia—the willingness to try new foods—and overall food satisfaction [40,43]. Despite these numerous studies on the factors that contribute to plate waste, there remains a significant gap in our understanding of the psychological influences on this behavior. In particular, we believe that further exploration of dispositional greed-an intense desire for resources-could provide valuable insights into the problem of plate waste. Thus, our study seeks to explore the role of dispositional greed in consumers’ plate-wasting behavior.

### 2.3. The Effect of Dispositional Greed on Plate Waste

While the factors outlined above provide a comprehensive view of the current literature on plate waste, our study suggests that additional, potentially overlooked, psychological elements may exacerbate the problem. Dispositional greed, characterized by an intense desire or hunger for resources [44,45], is one such factor. Traditionally viewed as a destructive force that promotes overconsumption and negatively impacts societal well-being [46], greed has been associated with serious outcomes such as wars, social and personal conflicts, and financial scandals [44,47]. However, its motivational power is also recognized as a driver of economic growth and development [48,49]. Indeed, greed is considered an inherent part of human nature, with all individuals possessing some degree of greed [45]. Although often associated with excessive desires for money or material goods, greed can extend to other domains, including power, status, sex, and, crucially for our study, food. These domains significantly influence consumers’ psychological and behavioral preferences [44,50]. Consequently, dispositional greed drives consumers to buy more food than they can consume. This inference arises from the observation that individuals with high levels of greed may not engage in rational cost-benefit assessments due to their overwhelming desire for more [51]. They may be susceptible to consuming disproportionately to their physiological hunger signals, influenced by the allure of food. Essentially, we propose that this psychological state, which is present in all individuals to some degree and goes beyond physiological hunger [45], could have a significant impact on plate waste. Based on these considerations, we propose the first hypothesis of our study:

**H1.**  *High levels of dispositional greed in consumers increase plate waste*.

### 2.4. The Link between Dispositional Greed and Impulsivity

The psychological literature has consistently revealed that greed often manifests itself in selfishness, self-seeking behavior, jealousy, and materialism [44]. Greed’s association with such tendencies may also promote less ethically sensitive actions and even unethical practices [52]. Further nuances of this trait have been observed in the form of low self-control and increased purchase impulsivity [53], suggesting a more complex interplay of dispositional traits. In particular, greedy individuals tend to exhibit a propensity for impulsive decision-making and often exhibit myopic behavior [54]. The term impulsivity deserves special attention here. It refers to an individual’s unplanned responses to stimuli in the absence of self-control or the ability to inhibit such responses [55]. In certain contexts, impulsivity is represented as “delay discounting,” in which the tendency to prefer an immediate, smaller reward overshadows the patience required to wait for a larger, delayed reward [56]. As a result, individuals characterized by impulsive personality traits may react immediately, irrationally, and without adequate consideration of the potential consequences of their actions.

The dynamics of greed and impulsivity have been examined in previous research, which has consistently shown a positive correlation between these constructs [45,53,57]. Interestingly, Seuntjens et al. [45] found that escalating levels of dispositional greed can increase impulsivity and thereby undermine self-control, even when impulsivity is not an explicit component of dispositional greed. Given these research findings, we propose the following hypothesis:

**H2.**  *Dispositional greed positively influences impulsivity*.

### 2.5. The Role of Impulsivity in Plate Waste

Impulsivity, often defined as an individual’s instantaneous response to internal and external stimuli without consideration of potential consequences [58], plays an important role in characterizing certain types of eating behaviors, including binge eating [59,60]. For example, external eating, which has been described as the consumption of food based on external cues such as how food looks or smells without physiological hunger [61], and overeating are associated with high impulsivity [62].

From this perspective, we believe that the impressive and diverse food choices offered by BSS trigger impulsivity as a strong external stimulus, which prepares the ground for individuals’ external eating and overeating tendencies when encountering BSS, as it may deviate them from rationality; thus, individuals may tend to take more food than they can consume. In addition, tourism experiences often produce a more positive mood for individuals compared to their everyday routines [63]. Intense positive moods reduce rationality and increase impulsive behavior [64], and positive moods increase food intake even in individuals of normal body weight [65]. In this case, we believe that plate waste will increase as individuals turn to consume foods they are not used to consuming because they are under the influence of impulsivity due to their intensely positive moods. Although direct links between impulsivity and plate waste have not been extensively explored in the existing literature, studies investigating impulsivity in the context of planned purchase behavior have found a positive correlation between impulsivity and increased food waste [66,67]. Based on these findings, we hypothesize that impulsivity, which weakens rational behavior and leads to myopic behavior [54], increases plate waste and propose the following H3 hypothesis.

**H3.**  *Increased levels of impulsivity in individuals increase the amount of plate waste*.

In addition, the literature has found positive associations between greedy personalities and impulsivity [53,57]. According to Seuntjens et al. [45], an escalation in dispositional greed increases impulsivity and thereby decreases self-control, even though impulsivity is not a well-defined component of dispositional greed. Based on this observation, we propose that impulsivity may play a mediating role in the relationship between dispositional greed and plate waste. This proposition constitutes our fourth hypothesis.

**H4.**  *Impulsivity mediates the relationship between dispositional greed and plate waste*.

### 2.6. Food Satisfaction and Ecolabeling as Moderators

Plate waste, which can be reduced by up to 92% without negatively affecting consumer satisfaction [4,41], represents a category of food waste that is relatively easy to manage [6]. In this context, it is an important focus of this study to investigate whether the variables of food satisfaction and ecolabeling play a significant role in reducing respondents’ plate waste. The concept of food satisfaction [68], which is defined as “a positive response to food after receiving it and food-related physical and psychological well-being sensations”, is influenced by a variety of factors, including traditional menus, authenticity, variety of cooking methods, unique flavors, originality, an affective image of food, an image of local cuisine, cultural heritage, uniqueness and price of food [69], its value, utility, appropriateness [70], diversity, and quality. However, the existing literature hardly covers the relationship between food satisfaction and plate waste. Some studies found no significant effect of food satisfaction on plate waste [71], while others found a decrease in plate waste with increasing food satisfaction [40]. Food quality, a determinant of food satisfaction, also indirectly affects plate waste by influencing consumers’ behavioral intentions [72,73,74,75,76].

Another important concept relevant to food waste is ecolabeling. Ecolabeling is the global practice of voluntary documentation and labeling of environmental performance [77] and has found its counterparts in tourism as well as other sectors [78,79]. According to Fairweather et al. [80], a tourism ecolabel is any form of certification that provides assurance that the tourism operation or activity is carried out according to a known standard that improves the environment or at least minimizes environmental impacts. Ecolabels are also important resources for informing consumers about the environmental impacts of the products or services they choose [81]. Although ecolabels are communication media that aim to internalize the external effects on the environment of the production, consumption, and disposal of products [82], they are still green marketing components that have been shown to influence the purchasing behavior of environmentally conscious tourists [83,84]. Moreover, ecolabels are directly related to both sustainable consumption and production [85,86] and food waste [87,88]. Ecolabels are expected to have a significant impact on food waste reduction. According to the EU Ecolabel [89], tourist accommodation establishments that are awarded the EU Ecolabel must develop a food waste management plan and monitor their food waste levels. In a study they conducted, Yılmaz et al. [90] found that sustainable management and administration activities were significantly different between hotels with and without an ecolabel. The study also concluded that accommodations that have ecolabel inform their consumers about the preservation of the environment and local cultures while developing and implementing policies to reduce the organization’s negative impact on the environment. Based on these findings in the literature, we believe that the effects of dispositional greed and impulsivity on plate waste will differ according to the hotels’ possession of ecolabel certificates and food satisfaction. Therefore, we propose the following hypotheses H5 and H6.

**H5.**  *The effect of impulsivity on plate waste is moderated by food satisfaction and ecolabel*.

**H6.**  *Food satisfaction and ecolabeling practice play moderating roles in the effect of dispositional greed on plate waste as mediated by impulsivity*.

## 3. Materials and Methods

### 3.1. Sampling and Data Collection

Southern Europe and Mediterranean countries attract the highest number of visitors according to international tourism mobility statistics [91]. For the countries in these regions and especially for Türkiye, Alanya is a popular destination for mass tourism with its trio of sea, sand, and sun (3S). Alanya attracted approximately 4.1 million tourists in 2022 [92,93]. Alanya is one of the top destinations in Türkiye, with the number of five-star hotels it has. The city is also distinguished by its number of five-star hotels, with 11% of Türkiye’s five-star hotels (n = 93) located in Alanya [21]. Also, many of the five-star hotels in Alanya have been implementing the all-inclusive holiday concept for a long time [94,95]. A total of 25 in 93 accommodation companies in Alanya possess The Green Star certification, indicating their environmental sensitivity. The Green Star certificate is granted by the Turkish Ministry of Culture and Tourism to protect the environment, raise environmental awareness, and encourage and support tourist facilities’ positive contributions to the environment within the scope of sustainable tourism. Reducing waste in hotels is also among the Green Star practices. They also include EU criteria (Ecolabels) [96]. Since Alanya is a top mass tourism destination in Türkiye, in addition to relying on the all-inclusive holiday concept and common adoptions of buffet service systems, this study is carried out in the region of Alanya.

Data for the study were collected through a questionnaire using a quantitative cross-sectional design. In order to ensure that tourists of different nationalities participated in the study, the questionnaires were prepared in German, Russian, English, and Turkish. The back-translation method was used to translate the questionnaires into the target languages [97]. Certified translators with advanced language skills in English, German, Russian, and Turkish were used in the translation process of the survey instruments. These translators were bilingual in the language pairs they translated and had professional experience in translation work. For the back-translation process, the translated versions of the surveys (in German, Russian, and Turkish) were independently translated back into English by a separate group of translators. These translators were also certified, experienced, and blind to the original English version of the survey. The purpose of the back-translation process was to verify the accuracy of the original translation and to ensure that the meaning of the items remained the same after translation. After back-translation, the original English version and the back-translated version were compared by the research team to ensure the consistency of the items. According to the checks, the translations were found to be consistent. Finally, the translated questionnaires were pre-tested on 40 tourists, evenly distributed among the four language groups, to verify comprehension. The pre-tests showed that all questions were well understood, and the questionnaires took their final forms as such.

In this study, the convenience sampling method from non-probability sampling methods was preferred. In the convenience sampling method, researchers include the units that they can easily access and select from the population [98]. First, the general managers of 49 five-star hotels in the region that could be contacted for the study were interviewed. The general managers were informed about the scope and content of the study. Of these, 14 five-star hotels with EL and 18 five-star hotels without EL agreed to participate in the study. The researchers first informed the hotel customers about the scope of the study and then distributed the questionnaires. Data were collected from 10 June 2022 to 20 October 2022. Participants who collected data in this study participated voluntarily. At the beginning of data collection, all participants were given an understandable explanation about the purpose of the research; it was explained to them that participation was voluntary, they could leave the process at any time without giving any reason, and they were assured of the anonymity and confidentiality of the data they provided. In this regard, all procedures involving human participants in the study were conducted in accordance with the ethical standards of the institutional and/or national research committee and the 1964 Declaration of Helsinki. Researchers strictly adhered to ethical principles (e.g., respect, autonomy, confidentiality, beneficence, and nonmaleficence) to ensure the integrity of the research. No experimental or clinical data were collected from participants.

### 3.2. Instrument

The constructs in this study were measured using multiple items. Respondents’ levels of dispositional greed were measured using the Dispositional Greed Scale (DGS), which was developed by Seuntjens et al. [44] and consisted of seven items. The scale has a single-factor design, with questions to be answered on a 7-point Likert scale (1 = strongly disagree; 7 = strongly agree). Consumers’ tendency to act impulsively was measured using the motor impulsivity factor propositions of the Abbreviated Impulsiveness Scale (ABIS) developed by Coutlee et al., [99]. Motor impulsivity refers to acting without thinking. A high score on this scale indicates that the consumer acts impulsively without thinking about the consequences of their actions. Motor impulsivity includes four statements, and each respondent was asked to indicate the frequency with which they experienced each statement. The items in the scale were measured using a 7-point Likert scale (1 = never, 7 = always). The respondents’ level of satisfaction with food was measured using seven statements compiled from the studies of Ryu et al. [100] and Yasami [101] et al. The scale measures the level of satisfaction with the food offered in hotels. Food satisfaction was measured using a 7-point Likert scale, ranging from (1) strongly disagree to (7) strongly agree. Respondents’ plate waste attitude was measured using four propositions prepared from the work of Tekin and Ilyasov [22]. Plate waste attitudes were measured with a 7-point Likert scale, ranging from (1) strongly disagree to (7) strongly agree. A high score on this scale indicates a high level of plate waste attitude among respondents. Face validity must also be revealed to prove the clarity of the statements in the compiled measurement tools [102]. Face validity refers to the ability of the scale to measure the item that is intended to be measured. To test the face validity of the scales, they were evaluated by two hospitality and tourism scholars and three food and beverage managers from different five-star hotels. They concluded that the statements in the scales are not problematic and that both academics and managers in the sector understand them in the same way. The demographic data of the respondents, such as age, gender, educational level, and occupation, were also collected using a demographic information form.

### 3.3. Data Analysis

**Figure 1:**
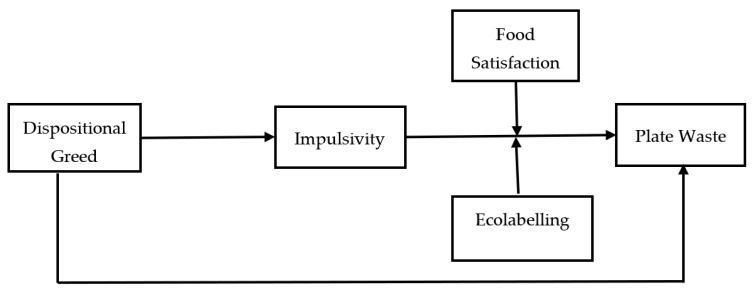
Research model for the study.

The moderated mediation research model presented in Figure 1 is tested in this study. The moderated mediation research model aims to explain how the mediating effect differs depending on the moderating variable(s). Prior to the analysis, the data were checked for suitability for multivariate statistical studies. First, missing values, extreme outliers, and normal distribution were checked. A total of 28 data with missing values greater than 5% were excluded. On the other hand, seven data were found to have a missing value below 5%. Little’s MCAR test (χ^2^ (105) = 119.14, *p* > 0.05) for these data indicated that the missing variables were random. Instead of these missing variables, data imputation was performed using the mean substitution method. Mahalanobis distance was used to detect outliers, and no outliers were found. Later, skewness and kurtosis coefficients were analyzed to verify the assumption of normal distribution. After the data-cleaning process, the research model analyses were conducted in two stages, as recommended by Anderson and Gerbing [103]. Confirmatory factor analysis (CFA) was used to test the measurement model in the first phase and the structural model in the second phase. The hypotheses of the study were tested using the PROCESS macro for SPSS models developed by Hayes [104].

## 4. Results

### 4.1. Demographic Findings

Information on respondent demographics is provided in Table 1. A total of 1253 respondents participated in the study; 61.2% were male and 38.8% were female. Most of the respondents (64.8%) were married. In terms of nationalities, 22.8% were Russian, 21.0% were German, and 18% were Turkish, for a total of 62% of all respondents. The remaining respondents were English (12.4%), Dutch (8.9%), Kazakh (7.7%), Polish (6.2%) and other countries (3.0%). The highest percentage of respondents (30.8%) had a college degree, followed by 27.9% with a bachelor’s degree and 24.4% with a high school diploma. An examination of the respondents’ professional backgrounds revealed that 65.8% worked in the private sector, 19.1% were civil servants, and 15.1% were self-employed.

### 4.2. Measurement Model

The measurement model, consisting of four constructs and 22 items (Table 2), showed an excellent fit in the confirmatory factor analysis (CFA). The model fit statistics indicated an acceptable model (χ^2^ = 449.870, df = 196, χ^2^/^df^ = 2.30, *p* < 0.001, RMSEA = 0.032, SRMR = 0.014, CFI = 0.993, IFI = 0.993, NFI = 0.987, RFI = 0.985), suggesting an overall robust model fit in accordance with the criteria recommended by Schermelleh-Engel et al. [105]. In terms of construct validity (Table 3), all constructs showed high internal reliability, with Cronbach’s alpha coefficients ranging from 0.93 to 0.97, and Composite Reliability (CR) and MaxR(H) values above the threshold of 0.70, indicating internal consistency [106]. Convergent validity was established as all Average Variance Extracted (AVE) scores were greater than 0.50, and CR scores exceeded AVE scores [107]. In terms of discriminant validity, according to the criteria of Fornell and Larcker [108], the square root of the AVE values of all constructs exceeded their respective correlations with other constructs. In addition, the AVE values exceeded the Maximum Shared Squared Variance (MSV) and Average Shared Squared Variance (ASV) values. In addition, all heterotrait-monotrait correlations (HTMT) were less than 0.90 [109], strengthening the discriminant validity. Our data distribution was found to be normal, with skewness values between 0.38 and −0.37 and kurtosis values between −1.44 and 0.40 [110]. Furthermore, the absence of multicollinearity was confirmed as the variance inflation factor (VIF) values were less than 5, and the tolerance values were greater than 0.30 [111]. These results substantiate the reliability and validity of our measurement model, thus ensuring its acceptability.

### 4.3. Common Method Bias

Common method bias (CMB) is a systematic error that can occur in research when the same method is used to measure multiple variables. More specifically, it refers to the variance that is attributed more to the measurement method than to the constructs being measured [112]. CMB can distort the estimated relationships between measures, obscuring true relationships or creating nonexistent ones [113]. Empirical studies are particularly susceptible to CMB when data are collected cross-sectionally from a single source [114]. When CMB is present, it can cause the observed correlations between variables to be larger or smaller than their true values [115]. In summary, the presence of CMB in the collected data may lead to errors in the analysis results of the proposed model between theoretical constructs [116]. To avoid CMB, we used a number of procedural and statistical methods. In a procedural manner, dependent, moderating, and independent variables were mixed in the questionnaire. At the same time, respondents were assured of the confidentiality of their answers. For the same reason, they were also asked not to include their identification or any other identifiable information about themselves on the questionnaires. Procedural measures were taken to reduce item priming effects among respondents [116]. For the statistical tests, the research model was compared to three alternative models using chi-square tests. As seen in Table 4, the four-factor research model was found to provide the best fit for the data. The results suggest that the hypothesized four-factor model provides the best fit to the data, mitigating the concerns of the CMB for this study.

### 4.4. Hypothesis Testing Results

First, the total, direct, and indirect effects on the relationship DISGRE→MPULS→ PLATEWASTE were tested without the moderator variables. According to the analysis results in Table 5 (Model 1), dispositional greed has a positive effect on plate waste in the absence of impulsivity (β = 0.50, t(1247) = 21.54, %95 CI [0.45; 0.54], *p* < 0.01). The results in Model 2 show the effect of dispositional greed, the independent variable of the study, on impulsivity, the mediating variable. Accordingly, dispositional greed has a positive effect on impulsivity (β = 0.76, t(1247) = 26.70, %95 CI [0.70; 0.81], *p* < 0.01). Model 3 shows the results of the mediator analysis revealing the effect of dispositional greed and impulsivity on plate wasting. Accordingly, dispositional greed (β = 0.31, t(1246) = 11.21, %95 CI [0.26; 0.36], *p* < 0.01) and impulsivity (β = 0.25, t(1246) = 11.31, %95 CI [0.21; 0.29], *p* < 0.01) positively influence plate wasting. It was also found that gender, age, and educational status, which were included as control variables in the model, did not significantly affect plate waste. The results of mediation analysis (model 3) showed that the indirect effect of dispositional greed on plate wasting via impulsivity (DISGRE → IMPULS → PLATEWST) was significant (β = 0.19%95 BCA CI [0.16; 0.22]). According to these results, hypotheses H1, H2, H3, and H4 are supported.

To test how the effect of impulsivity on plate waste differed according to food satisfaction and hotel ecolabeling, we performed an additive multiple moderation analysis. We also included gender, age, and education as covariates in the model. According to the results in Table 5 Model 4, all the variables included in the model explain 57% of the change in plate waste. The interaction effect of impulsivity and food satisfaction on plate waste (β = −0.08, t(1243) = −8.70, %95 CI [−0.09; −0.05], *p* < 0.01) was found to be statistically significant, while the interaction effect of impulsivity and hotel ecolabeling status (β = −0.03, t(1243) = −1.22, %95 CI [−0.11; 0.03], *p* > 0.05) was not statistically significant. According to these results, the effect of impulsivity on plate waste differs only by food satisfaction.

The conditional effect on the values of the moderators is shown in Table 6. The details of the conditional effects show that consumers’ level of plate waste increases significantly as their level of food satisfaction decreases. Although plate waste is lower in hotels that have an ecolabel, the presence of the ecolabel does not make a statistically significant difference in the amount of plate waste. The effect of impulsivity on plate waste is strongest in hotels without an ecolabel and where consumers have low food satisfaction (β = 0.51, %95 CI [0.46; 0.57], *p* < 0.01). On the other hand, hotels with an ecolabel, whose consumers have a high level of food satisfaction, show that the effect of impulsivity on plate waste decreases to the lowest levels (β = 0.23, %95 CI [0.16; 0.30], *p* < 0.01). These results empirically demonstrate the importance of food satisfaction in reducing the effect of impulsivity on plate waste. No statistically significant effect of ecolabeling was found in reducing the effect of impulsivity on plate waste. Therefore, hypothesis H5 was not supported.

Finally, the moderated mediation model was tested in the study, the results of which are presented in Table 7. The index of multiple moderated mediation of food satisfaction was significant (β = −0.05, 95% CI [−0.06, −0.03]), while the index of multiple moderated mediation of ecolabel was not significant (β = −0.03, 95% CI [−0.08, 0.02]). These results indicate that food satisfaction is a moderating variable for the indirect effect of dispositional greed on plate waste via impulsivity. The conditional indirect effects in Table 6 show that food satisfaction has a much stronger effect than ecolabeling in reducing the effect of dispositional greed on plate waste via impulsivity.

## 5. Discussion

Many of the hotels in the destination in which this study was conducted have been providing services with the all-inclusive holiday concept for a long time [94,95]. Foods in an all-inclusive holiday concept are commonly presented with a buffet service system. Since it is almost impossible to create a single menu that would appeal to the tastes of all consumers, the buffet service system became more popular because it offers consumers several options at the same time and without portion limits [117]. However, the impressive offering of many foods without portion control psychologically influences consumers in ways that would diverge them from rational consumer behavior, leading them to take more food than they can consume [118,119]. Consequently, plate waste increase with the buffet service system is an important and underlying factor [4,23,24]. Our study aimed to examine the interplay of dispositional greed, impulsivity, food satisfaction, and ecolabeling on consumers’ plate waste in the context of an all-inclusive buffet system. The main findings show that dispositional greed and impulsivity significantly increase the problem of consumer plate waste. In contrast, ecolabels were found to have no significant effect in mitigating the effects of greed and impulsivity on plate waste. However, the role of food satisfaction was found to be highly significant in this context. These results are discussed and analyzed in the context of the literature.

Food in an all-inclusive holiday concept is often presented through a buffet service system. Although the broad food offerings of the buffet system are intended to cater to diverse tastes [117], this lack of portion control may lead consumers to deviate from rational consumption patterns, resulting in increased plate waste [118,119]. Characteristics inherent to greedy individuals, such as difficulty setting limits and satiation levels [44], are likely to amplify this phenomenon. The stimulating atmosphere of the open buffet, coupled with the predisposition towards greed, could result in individuals serving themselves more food than they can consume. Our results corroborate these assumptions, solidifying the role of dispositional greed as a major factor leading to increased plate waste.

Consistent with previous studies [45,53,57], we found a positive correlation between dispositional greed and impulsivity. Greedy people are especially impulsive decision-makers [57]. They act with lower levels of self-control due to impulsivity and cannot contain themselves [55]; when they are combined with the impressive atmosphere of the buffet service system, the proper ground for the interaction between dispositional greed and impulsivity is likely to be set up. On the other hand, another finding of this study was impulsivity being an effective factor in increasing plate waste, much like dispositional greed. Many experts highlight that the main reason for waste in food consumption on a consumer level is due to the lack of planning and management [120], drawing attention to impulsive behavior that occurs in an unplanned and uncontrolled manner. In addition, “consumption culture”, in combination with the factor of food abundance, may cause people to overlook the potential food waste with the influence of impulsivity [121]. In this context, we believe that the buffet service system causes consumers to react impulsively to external stimuli from foods due to the impressive atmosphere it provides, leading to the perception of food abundance without thinking about the consequences [58], which causes consumers to take more food onto their plates than they can consume in ways that are disproportionate with their physiological hunger. Therefore, our findings argue for the significant contribution of both dispositional greed and impulsivity to the plate waste issue.

According to the other results of the study, we found that food satisfaction has a significant effect on consumers’ plate waste, but the effect of ecolabeling is not statistically significant. In short, we have come up with two striking results. First, food satisfaction appears to be a critical factor that may be able to reduce the effect of dispositional greed and impulsivity on plate waste. This is consistent with research showing that increased food satisfaction and quality can reduce food waste [40,73,74,75,76,122,123]. The second striking result in our study was that contrary to what was expected, ecolabelling surprisingly had no significant effects on the decrease of plate waste. According to the findings, plate waste is lower in hotels that have ecolabel, but this difference is not statistically significant. This finding indicates that ecolabels’ behavioral impact on consumers is insufficient in decreasing plate waste. According to Cerqua [124], scientists did not reach a consensus on the efficacy of ecolabels in improving environmental conditions. Various studies have also revealed the positive impact of ecolabels on consumer behavior [77]. However, Buckley [78] claims that ecolabels are green marketing tools, and similarly, Reiser and Simmons [125] posit that positive attitudes of tourists towards sustainability labels such as ecolabels are not enough to prove their environmentalist behaviors. Insufficient and unenlightening information and messages about ecolabels [126,127] and their negatively framed forms [128] decrease ecolabels’ impacts on consumer behavior. In this context, we have concluded in this study that even though ecolabels raise awareness, they are not sufficiently effective on plate waste; only food satisfaction is a moderating variable on the indirect effect of dispositional greed on plate waste via impulsivity.

### Theoretical and Practical Implications

This study provides both theoretical and practical implications for researchers, policymakers, and industry professionals working to mitigate food waste in the tourism and hospitality sector. On the theoretical side, our findings contribute to the emerging literature that examines the interplay of dispositional greed, impulsivity, food satisfaction, and ecolabeling on consumer plate waste in the buffet system environment. This study extends the existing knowledge by revealing the relative importance of these variables on plate waste, particularly highlighting the crucial role of dispositional greed and impulsivity. We emphasize the need to consider these psychological factors when modeling consumer behavior related to plate waste. In addition, the study challenges the presumed efficacy of ecolabels in reducing plate waste and underscores the need for further research to identify the conditions under which ecolabels may have a significant impact on consumer behavior.

From a practical standpoint, the study offers meaningful implications for industry practitioners, particularly in hotels and similar establishments offering buffet services. The results suggest that attention should be focused on improving food satisfaction, as this variable has a significant impact on plate waste. Strategies such as optimizing menu variety, improving food quality, or providing personalized meal recommendations could be employed to this end. Furthermore, our study calls for more robust strategies to increase the impact of ecolabels. Awareness campaigns that explain the environmental benefits of reducing plate waste, coupled with positively framed messages about the hotel’s ecolabel status, could strengthen the impact of ecolabels on plate waste.

## 6. Conclusions

This study concluded that dispositional greed and impulsivity factors significantly influence consumers’ plate waste. Interestingly, while ecolabels had no significant effect on the impact of dispositional greed and impulsivity on plate waste, nor on plate waste reduction, the role of food satisfaction was found to be critically important. In light of these findings, we believe that tourism and hospitality organizations need to pay special attention to food satisfaction as a strategy to reduce plate waste. This includes the regular and systematic measurement of food satisfaction along with plate waste levels, allowing for the control and monitoring of consumer food satisfaction levels and associated plate waste levels. Despite the observed minimal impact of ecolabels, the findings suggest that ecolabeling organizations should implement robust awareness campaigns. This would enhance the potential positive impact of ecolabels on plate waste, particularly if these labels are presented with more compelling, positive messages. Future research is encouraged to explore strategies to counteract dispositional factors of greed and impulsivity, given the significant influence of these factors on plate waste, as revealed in our study. Thus, the development of methods to reduce dispositional greed and impulsivity may provide valuable psychological tools to combat plate waste. As research on the psychological elements and drivers of plate waste behavior increases, we anticipate that effective psychological interventions can be developed to reduce plate waste behavior without negatively impacting consumer satisfaction.

## Figures and Tables

**Table 1:** Demographic profile of respondents (n = 1253).

Demographics	Groups	n	%	Demographics	Groups	n	%
Gender	Male	766	61.2	Age	18–28	351	28.0
	Female	486	38.8		26–33	387	30.9
Marital status	Single	441	35.2		34–41	289	23.1
	Married	811	64.8		42–49	154	12.3
Nationality	Russian	286	22.8		≥50	71	5.7
	German	263	21.0	Education	Primary	116	9.3
	Turkish	225	18.0		High	306	24.4
	English	155	12.4		College	385	30.8
	Dutch	112	8.9		Bachelor’s degree	349	27.9
	Kazakh	96	7.7		Post-graduate	96	7.7
	Polish	78	6.2	Employed sector	Government	239	19.1
	Other	37	3.0		Private sector	824	65.8
					Own business	189	15.1

**Table 2:** Result of measurement model.

Construct	Items	Mean	SD	Factor Loadings	*t*-Value
Dispositional Greed	I always want more	3.09	1.40	0.868	Fixed
	Actually, I’m kind of greedy	3.16	1.42	0.875	38.09 ***
	One can never have too much money (R)	3.20	1.55	0.871	42.82 ***
	As soon as I have acquired something, I start to think about the next thing I want	3.21	1.54	0.892	45.27 ***
	It doesn’t matter how much I have, I’m never completely satisfied	3.18	1.54	0.890	45.29 ***
	My life motto is ‘more is better’	3.17	1.56	0.921	48.78 ***
	I can’t imagine having too many things (R)	3.13	1.57	0.935	50.48 ***
Impulsivity	I act on impulse	3.37	1.89	0.899	Fixed
	I do things without thinking	3.25	1.74	0.916	63.97 ***
	I say things without thinking	3.31	1.81	0.919	52.29 ***
	I act on the spur of the moment	3.34	1.89	0.952	57.32 ***
Food Satisfaction	The food was delicious	4.49	1.76	0.914	Fixed
	The restaurant offered fresh food	4.46	1.83	0.898	52.56 ***
	The smell of the food was enticing	4.58	1.92	0.909	54.24 ***
	The food presentation was visually attractive	4.60	1.86	0.929	58.11 ***
	The restaurant offered a variety of food.	4.59	1.92	0.926	57.75 ***
	Food is served at the appropriate temperature	4.58	1.92	0.935	59.91 ***
	All food service areas and surfaces were clean	4.53	1.90	0.950	63.41 ***
Plate Waste	I could not finish all the food I have put on my plate.	3.20	1.52	0.932	Fixed
	I have put food on plate in amounts I can consume.	3.17	1.44	0.856	46.50 ***
	I have tried to finish all the food I have put on my plate.	3.27	1.48	0.870	49.13 ***
	I did not leave any leftovers from the food I have put on my plate.	3.20	1.48	0.888	51.13 ***

*** *p* < 0.001, Responses are on a 7-point Likert scale.

**Table 3:** Results of discriminant and convergent validity.

	α	MaxR(H)	CR	AVE	MSV	ASV	1	2	3	4
1. DISGRE	0.96	0.97	0.96	0.80	0.39	0.23	[0.89]			
2. IMPUL	0.96	0.96	0.96	0.85	0.39	0.24	0.64 **	[0.92]		
3. FOODSAT	0.97	0.98	0.98	0.85	0.28	0.10	−0.18 **	0.04	[0.92]	
4. PLTWST	0.93	0.94	0.94	0.79	0.32	0.29	0.54 **	0.59 **	−0.55 **	[0.89]

DISGRE = Dispositional Greed, IMPUL = Impulsivity, FOODSAT = Food Satisfaction, PLTWST = Plate Waste, α = Cronbach’s Alpha, CR = Composite Reliability, AVE = Average Variance Extracted, ASV = Average Shared Variance, MSV = Maximum Shared Variance, α = Cronbach Alfa, Values in square brackets [] are the square root values of AVE, ** *p* < 0.001.

**Table 4:** Comparison of alternative measurement models for main constructs.

Models	χ²	df	χ²/df	CFI	RMSEA	Model Comparison	∆χ²	∆df	p (∆χ²)
1. Hypothesized four-factor model ^a^	449.87	196	2.30	0.993	0.032		-	-	
2. Three-factor model ^b^	3620.1	199	18.19	0.9	0.117	2 vs. 1	3170.21	3	0.01
3. Two-factor Model ^c^	13,519	201	67.26	0.612	0.23	3 vs. 1	13,068.90	5	0.01
4. One-factor Model ^d^	16,720	202	82.77	0.518	0.256	4 vs. 1	16,269.95	6	0.01

^a^ = Dispositional Greed; Impulsivity; Food Satisfaction; Plate Waste, ^b^ = Dispositional Greed + Impulsivity; Food Satisfaction; Plate Waste, ^c^ = Dispositional Greed + Impulsivity + Food Satisfaction; Plate Waste, ^d^ = Dispositional Greed + Impulsivity + Food Satisfaction+ Plate Waste.

**Table 5:** Results for testing hypotheses.

	Relations	β	SE	t	LLCI	ULCI
Model 1	DISGRE	0.50	0.02	21.54 *	0.45	0.54
	GENDER	0.04	0.07	0.61	−0.09	0.18
	AGE	0.01	0.03	0.44	−0.04	0.07
	EDU	−0.02	0.03	−0.57	−0.08	0.04
	R^2^ = 0.27, F_(4.1247)_ = 116.548, *p* < 0.001					
Model 2	DISGRE	0.76	0.03	26.70 *	0.70	0.81
	GENDER	−0.07	0.08	−0.86	−0.24	0.09
	AGE	0.00	0.04	0.04	−0.07	0.07
	EDU	−0.02	0.04	−0.56	−0.09	0.05
	R^2^ = 0.37, F_(4.1247)_ = 179.243, *p* < 0.001					
Model 3	DISGRE	0.31	0.03	11.21 *	0.26	0.36
	IMPULS	0.25	0.02	11.31 *	0.21	0.29
	GENDER	0.06	0.07	0.91	−0.07	0.19
	AGE	0.01	0.03	0.45	−0.04	0.07
	EDU	−0.01	0.03	−0.42	−0.07	0.05
	Bootstrap Indirect Effect					
	DISGRE → IMPULS → PLTWST	0.19	0.02	-	0.16	0.22
	R^2^ = 0.34, F_(5.1246)_ = 128.330, *p* < 0.001					
Model 4	IMPULS	0.38	0.01	26.89 *	0.33	0.41
	FOOSAT	−0.41	0.02	−22.20 *	−0.45	−0.38
	IMPULS × FOOSAT	−0.08	0.01	−8.70 *	−0.09	−0.05
	ECOLBL	−0.19	0.07	−2.82 *	−0.31	−0.06
	IMPULS × ECOLBL	−0.03	0.03	−0.74	−0.09	0.04
	GENDER	0.05	0.05	0.96	−0.05	0.15
	AGE	0.02	0.02	0.06	−0.03	0.06
	EDU	0.02	0.02	1.22	−0.07	0.02
	R^2^ = 0.57, F_(8.1243)_ = 212.771, *p* < 0.001					
Model 5	DISGRE	0.16	0.02	7.03 *	0.12	0.20
	IMPULS	0.30	0.02	17.34 *	0.27	0.34
	FOOSAT	−0.39	0.02	−21.18 *	−0.43	−0.36
	IMPULS × FOOSAT	−0.07	0.01	−7.52 *	−0.08	−0.05
	ECOLBL	−0.19	0.06	−2.99 *	−0.32	−0.07
	IMPULS × ECOLBL	−0.04	0.03	−1.22	−0.11	0.03
	GENDER	0.05	0.05	1.02	−0.05	0.15
	AGE	0.01	0.02	0.56	−0.03	0.05
	EDU	−0.02	0.02	−1.05	−0.07	0.02
	R^2^ = 0.59, F_(9.1242)_ = 201.979, *p* < 0.001					

* *p* < 0.01; DISGRE = Dispositional Greed, IMPUL = Impulsivity, FOODSAT = Food Satisfaction, PLTWST = Plate Waste.

**Table 6:** Conditional effects of impulsivity at values of the moderators.

Food Satisfaction	Ecolabel	β	SE	t	LLCI	ULCI
Low	No	0.51	0.03	18.904 ***	0.46	0.57
Low	Yes	0.49	0.03	19.685 ***	0.44	0.54
High	No	0.26	0.02	12.448 ***	0.21	0.30
High	Yes	0.23	0.03	6.940 ***	0.16	0.30

*** *p* < 0.001.

**Table 7:** Conditional indirect effects of dispositional greed on plate waste.

Food Satisfaction	Ecolabel	β	SE	LLCI	ULCI
Low	No	0.33	0.03	0.28	0.38
Low	Yes	0.29	0.02	0.25	0.34
High	No	0.16	0.02	0.13	0.19
High	Yes	0.13	0.03	0.07	0.18
Indices of partial moderated mediation:		β	SE	LLCI	ULCI
Food Satisfaction		−0.05	0.01	−0.06	−0.04
Ecolabel		−0.03	−0.03	−0.09	0.02

## Data Availability

The data presented in this study are available in anonymized form upon request from the corresponding author.
